# Demographic Profile, Health, and Associated Factors of Family Caregivers and Functionality of Hospitalized Older Adults: Cross-Sectional, Exploratory, and Descriptive Study

**DOI:** 10.2196/54074

**Published:** 2024-06-21

**Authors:** Mateus Cunha Gomes, Robert Castro, Willian Silva Serra, Jhak Sagica de Vasconcelos, Andressa Parente, Eliã Pinheiro Botelho, Glenda Ferreira, Fabianne Sousa

**Affiliations:** 1 Nursing School Federal University of Para Belem Brazil

**Keywords:** family caregiver, older adult, hospitalization, functionality, caregiver, health

## Abstract

**Background:**

The longevity of the world population can contribute to an increase in hospitalizations and, consequently, to the emergence of functional limitations, resulting in the need for family caregivers. Hospitalized older adults may become dependent and require more care, increasing the burden on family caregivers. Thus, the nursing team in the hospital environment faces a new situation: an increase in the number of older adults occupying hospital beds and the presence of their family caregivers.

**Objective:**

We aimed to analyze the association between the demographic variables of interest and the self-rated health of family caregivers and to describe the functionality of older adults hospitalized in a university hospital in the Amazonian context.

**Methods:**

This cross-sectional, quantitative, exploratory, and descriptive study was carried out through individual interviews with 98 interviewees, divided into 49 family caregivers and 49 older adults hospitalized in the surgical clinic sector of a university hospital in Brazil between February and March 2023. Demographic data and health conditions were collected from family caregivers, and to describe the functionality of hospitalized older adults, the Barthel Index was applied. Descriptive (frequency and percentage) and inferential analyses were used, and the student *t* test was applied. The significance level of 5% was adopted.

**Results:**

Among the 49 family caregivers, the majority were women (n=40, 81.6%) with an average age of 46.9 (SD 13.3) years. Most were single (n=28, 57.1%) and had completed an average level of education (n=26, 53.1%). Additionally, 25 (51%) caregivers were caring for their parents. Regarding health conditions, respondents self-assessed their health as good (25/49, 51%; *P*=.01), and they considered that their health status was not affected by the provision of care (36/49, 73.5%; *P*=.01). There was a significant association between demographic variables (ie, gender, age, and education) and self-assessment of family caregivers (*P*=.01, *P*=.01, and *P*=.04, respectively). Of the 49 older adults hospitalized, the majority (n=31, 63.2%) were men, with a mean age of 69.2 (SD 7.12) years. Regarding the assessment of functionality, most older adults were classified as having mild dependence on care (n=23, 46.9%), specifically in the age group between 60 and 69 years (21/49, 67.8%).

**Conclusions:**

The data revealed that female gender, age, and education of family caregivers contributed favorably to the provision of care to hospitalized older adults with a lower degree of functional dependence. It is important to emphasize that during the older adult’s hospitalization, the family caregiver should not be seen as a delegation of responsibilities or as a complement of human resources to assist in their recovery. Health professionals need to implement assertive interventions so that the family caregiver functions as a therapeutic resource.

## Introduction

Brazil has more than 30 million older adults, reaching 14% of the total population [1]. Brazilian projections show that, in 2030, the number of older adults will exceed that of children and adolescents aged 0 to 14 years by approximately 2.28 million, on the way to becoming a country with a majority older adult population [2].

In this way, there is a change in the population’s epidemiological profile; consequently, maintaining independence and autonomy is a challenge for this group, as they are more susceptible to chronic noncommunicable diseases, disabling conditions, sensory decline, accidents, and social isolation, requiring the help of caregivers for long periods [3].

In turn, the impairment of the functional capacity of older adults has a major impact on the lives of family members, caregivers, the health system, and mainly, on their own lives, as they need their functionality to play their roles in society and in daily activities. Not being functionally well means greater expenses, greater vulnerability, and dependence, reducing well-being and quality of life. Currently, the health of older adults is measured not by the number of diseases but by their functional capacity [4].

Therefore, it is essential that older adults, especially those hospitalized, have a comprehensive care support network, where the family plays a crucial role in guaranteeing their well-being and care, represented by the role of the family caregiver or informal caregiver [5].

The term “caregiver” is classified into formal and informal (family) caregivers. The formal caregiver is conceptualized as a technically qualified individual, who has experience in providing care at home and receives remuneration through a formal employment contract [6]. The family caregiver is defined as an individual who does not have professional training, works voluntarily, and provides assistance to an acquaintance or family member, such as a spouse, parents, or children [7], and is the most common form of assistance to older people [6,8].

The role of the caregiver involves perceiving the other person as an integral being, monitoring their daily activities, providing emotional and psychological support, and managing the finances of the individual they care for [2]. It should be noted that family caregivers have an important responsibility for patient care and often face challenges due to a lack of preparation for the situation, whether in terms of assistance, care management, or emotional support [9].

In Brazil, the Ministry of Health ensures the presence of a caregiver during the older adults’ hospitalization process, recognizing the improvement in their quality of life provided by caregiving; furthermore, it mandates provisions that facilitate caregivers’ presence and guarantees financial resources for their accommodation. Health care professionals are required to improve the care of older adults in partnership with the family caregiver [10].

International literature shows that high-complexity hospitals in South America, Europe, and Asia report that most older adults occupying the beds are women and have previous hospitalization experience. In Brazil, according to the Elderly Statute created by law number 10.741 on October 1, 2003, hospitalized older people are guaranteed the right to have a full-time companion [11].

In the hospital context, the presence of a family caregiver accompanying older adults has become an increasingly frequent phenomenon. It is noteworthy that functional dependence of older adults can be a stressful factor for family caregivers, as it compromises their well-being and quality of life, negatively impacting the quality of care offered to dependent older adults [12,13].

Studies indicate that the analysis between family caregivers and the functionality of older adults in the hospital context, especially in the Amazonian context, is still scarce.

Therefore, aiming to guide actions that promote the quality of care provided by family caregivers, this study aimed to analyze the association between demographic variables of interest and the self-rated health of family caregivers; additionally, it aimed to describe the functionality of older adults hospitalized in a university hospital in the Amazonian context.

## Methods

### Study Design

This was a cross-sectional, quantitative, descriptive, and observational study. in addition, the STROBE (Strengthening the Reporting of Observational Studies in Epidemiology) checklist for observational studies was used to help conduct the research and report the results obtained.

### Location and Period

This study was carried out in a surgical hospitalization unit of the João de Barros Barreto University Hospital Complex (HUJBB) in the city of Belém, Pará, Brazil. HUJBB is a highly complex hospital with 261 beds, a reference in the care of infectious and contagious diseases and oncology, with care in several specialties, including geriatrics, endocrinology, chemotherapy, radiotherapy, and general surgery.

The city of Belém is the capital of the state of Pará, which together with the states of Maranhão, Amapá, Tocantins, and Mato Grosso make up the Eastern Amazon.

Data collection took place between February 1 and March 30, 2023, in the hospital environment, specifically in the surgical hospitalization clinic—aimed at clinically stable pre- or postoperative (mediate and immediate) patients.

### Population, Sample, Inclusion Criteria, and Exclusion Criteria

Initially, a survey was conducted by the nursing leader of the inpatient unit, specifically the surgical clinic of this hospital, where an average of 15 to 20 caregivers pass through the unit per month.

The intentional and convenience nonrandom sampling technique was used to obtain the sample population.

All family caregivers who accompanied older adults hospitalized in the HUJBB surgical hospitalization unit during the data collection period were interviewed, totaling 98 interviewees. This group was divided into 49 family caregivers and 49 older adults hospitalized in the surgical clinic. We emphasize that there was no withdrawal or refusal by the interviewees to participate in the study.

The following were considered as inclusion criteria:

- Family caregivers: (1) aged 18 years and older, (2) ability to answer the questions on the questionnaires, (3) not being paid for providing care, and (4) availability of time to answer the research questionnaires.
- Older adults: (1) aged 60 years and older, (2) being hospitalized accompanied by an informal caregiver, (3) ability to answer the questionnaires’ questions (ie, stable clinical condition), (4) availability of time to answer the research questionnaires, and (5) receiving medical treatment for any acute or chronic illnesses.

The exclusion criteria were as follows:

- Family caregivers: (1) unable to respond to the questionnaires for any reason and (2) remunerated for the care provided.
- Older adults: (1) difficulty in understanding the questions or having cognitive disabilities, (2) altered clinical condition for any reason (eg, hypertension, hyper or hypoglycemia, and disorientation in time or space according to the evaluation criteria of health care professionals health), or (3) unable to complete the answers to the questions in the questionnaires.

### Data Collection Procedures and Instruments

Initially, the main researcher joined the team of professionals who were part of the surgical clinic to learn about the work routine in the research field.

The two following instruments were used:

1. A questionnaire with sociodemographic questions (eg, age, gender, marital status, education, and whether the caregiver lived with the older adult) and health-related questions, including health status, whether the caregiver’s health was affected by the provision of care, whether the caregiver was responsible for the care of another older person, and the degree of kinship.
2. The Barthel Index instrument to describe the functionality of older adults.

The Barthel Index is an instrument used worldwide to assess functional independence and mobility, presenting very consistent reliability and validity results with a Cronbach α value of 0.90. This was validated for use in Brazil; it evaluates 10 basic activities of daily living, with scores ranging from 0 to 100. A person with scores below 25 is considered completely dependent; scores from 50 to 26 indicate severe dependence; scores from 51 to 75 indicate moderate dependence; scores from 76 to 99 indicate mild dependence, and a score of 100 means complete independence [14].

Interviews were conducted by the main researcher with an average duration of 20 minutes, in different shifts (morning or afternoon) in the hospitalized older adult’s own room to promote privacy and confidentiality of information. As this was done during the COVID-19 pandemic period, the World Health Organization recommendations regarding the use of masks, minimum social distancing of 1 meter, and hand hygiene were followed.

### Statistical Analysis

The data were double entered into a Microsoft Excel spreadsheet and analyzed using the SPSS software for macOS (version 28.0; IBM Corp). In the descriptive analysis, the following were calculated: mean (SD) for quantitative variables as well as the absolute and relative frequencies. The Kolmogorov-Smirnov test was used to test the normality of the variables. Considering the lack of normality in the distribution of variables, the parametric Student *t* test (1-tailed) was applied; the choice to use this test was due to the fact that the sample was small. The level of significance adopted was *P*<.05.

### Ethical Considerations

The study followed the standards of the 2013 Declaration of Helsinki, ensuring confidentiality and anonymity of data of all participants, with a favorable opinion by the Ethics Committee on Human Research of the Federal University of Pará in accordance with resolution number 466/2012 of the National Health Council (CNS in Portuguese) receiving approval (5.312.450). All interviewees who agreed to participate in the research were presented with the objectives, risks, and benefits of the study; after providing them with this information, they signed the Free and Informed Consent Form, received a copy of it, and proceeded with the interview.

## Results

Of the 49 family caregivers interviewed, 40 (81.6%) were women, and 25 (51%) were aged between 41 and 59 years, with an average age of 46.9 (SD 13.3) years. Regarding their marital statuses, 28 (57.1%) were single, 26 (53.1%) had completed their high school education, and 25 (51%) had children. All results showed a statistically significant difference (*P*<.01; Table 1).

As shown in Table 2, a total of 25 (51%) caregivers self-assessed their health as good; 36 (73.5%) reported that their health was not affected by providing care to hospitalized older adults. Of the total number of caregivers, 27 (55.1%) provided care for 1 person. All results showed a statistically significant difference (*P*<.01).

Table 3 shows a bivariate analysis of the variables associated with self-assessment of the health status of family caregivers. There was a significant statistical association between the demographic variables (ie, gender, age, and education) and the respondents’ self-assessed health statuses (*P*<.001).

Of the 49 hospitalized older adults interviewed, 31 (63%) were men, and 19 (61%) were between 60 and 69 years of age. The average age was 69.2 (SD 7.12) years. The age group between 60 and 69 years had a degree of dependence between mild and independent (Table 4).

Regarding the functionality of hospitalized older adults who were interviewed, there was a predominance of older adults with a mild degree of dependence 23 (46.9%). All results were statistically significant (*P*<.01; Table 5).

**Table 1:** Demographic characteristics of family caregivers of older people hospitalized in a university hospital in Belém, Pará, Brazil, in 2023 (N=49).

Variables		Values, n (%)	*P* value^a^	
**Age (years)^b^**				.001
	22-40	15 (30)		
	41-59	25 (51)		
	≥60	9 (19)		
**Gender**				.001
	Female	40 (82)		
	Male	9 (18)		
**Marital status**				.001
	Married/stable union	16 (33)		
	Divorced/separated	1 (2)		
	Single	28 (57)		
	Widowed	4 (8)		
**Education**				.001
	Complete primary education	3 (6)		
	Incomplete elementary education	10 (20)		
	Complete high school	26 (53)		
	Complete higher education	9 (18)		
	Incomplete higher education	1 (2)		
**Relationship with the person you are the caregiver for**				.001
	Spouse/partner	8 (16)		
	Children	25 (51)		
	Friend/neighbor	5 (10)		
	Brother or brother-in-law	4 (8)		
	Grandson	6 (12)		
	Father/mother	1 (2)		

^a^Significant at *P*<.05 (student *t* test)*.*

^b^The mean age was 46.9 (SD 13.3) years.

**Table 2:** Description of the health conditions of family caregivers of older adults hospitalized in a university hospital in Belém, Pará, Brazil, in 2023 (N=49).

Variables			Values, n (%)		*P* value^a^	
**Health self-assessment**						<.001
	Good	25 (51)				
	Very good	5 (10)				
	Too bad	1 (2)				
	Neither good nor bad	17 (35)				
	Bad	1 (2)				
**Do you consider that your health status has been affected by the provision of care?**						.002
	No	36 (74)				
	Yes, negatively affected	13 (26)				
**How many people regularly care for and/or support them in their daily activities, personal care, or in other ways due to physical or mental illness, disability or old age?**						<.001
	1 person	27 (55)				
	2 people	13 (26)				
	3 people	4 (8)				
	More than 3 people	5 (10)				

^a^Significant at *P*<.05 (student *t* test).

**Table 3:** Bivariate analysis of variables associated with informal caregivers’ self-assessed health status in Belém, Pará, Brazil, in 2023.

Variables (N=49)		Self-evaluation: good (n=25), n (%)	Self-evaluation: very good (n=5), n (%)	Self-evaluation: neither good nor bad (n=17), n (%)	Self-evaluation: too bad/bad, (n=2), n (%)	*P* value^a^	
**Gender**							<.001
	Male	5 (20)	2 (40)	2 (12)	0 (0)		
	Female	20 (80)	3 (60)	15 (88)	2 (100)		
**Age (years)**							<.001
	22-40	9 (36)	2 (40)	4 (23)	0 (0)		
	41-59	12 (48)	2 (40)	10 (59)	1 (50)		
	≥60	4 (16)	1 (20)	3 (18)	1 (50)		
**Education**							.047
	Complete primary education	2 (8)	0 (0)	1(6)	0 (0)		
	Incomplete elementary education	5 (20)	1(20)	3(18)	1 (50)		
	Complete high school	11 (44)	3 (60)	11(65)	1 (50)		
	Complete higher education	7 (28)	1(20)	1 (6)	0 (0)		
	Incomplete higher education	0 (0)	0 (0)	1 (6)	0 (0)		
**Marital status**							.07
	Married/stable union	6 (24)	1(20)	8(47)	1 (50)		
	Divorced/separated	1 (4)	0 (0)	0 (0)	0 (0)		
	Single	16 (64)	4 (80)	8 (47)	0 (0)		
	Widower	2 (8)	0 (0)	1 (6)	1 (50)		

^a^Significant at *P*<.05 (student *t* test).

**Table 4:** Comparison between the degree of functionality and age with age group of older adults hospitalized in a university hospital. Belém, Pará, Brazil, (N=49), 2023.

Variables		Total sample (N=49, 100%), n (%)	Aged 60-69 years (n=31, 63%), n (%)	Aged 70-79 years (n=13, 27%), n (%)	Aged ≥80 years (n=5, 10%), n (%)	*P* value^a^	
**Degree of functionality**							.004
	Total/severe/moderate	16 (33)	10 (32)	3 (23)	3 (60)		
	Light/independent	33 (67)	21 (68)	10 (77)	2 (40)		
**Gender**							.004
	Male	31 (63)	19 (61)	8 (62)	1 (20)		
	Female	18 (37)	12 (39)	5 (38)	4 (80)		

^a^Significant at *P*<.05 (student *t* test)*.*

**Table 5:** Functionality classification of older adults hospitalized in a university hospital in Belém, Pará, Brazil, in 2023 (N=49).

Degree of functionality	Values, n (%)	*P* value^a^
Total dependence	5 (10)	<.001
Severe dependence	5 (10)	<.001
Moderate dependence	6 (12)	<.001
Light dependence	23 (47)	<.001
Total independence	10 (20)	<.001

^a^Significant at *P*<.05 (student *t* test).

## Discussion

### Principal Findings

The increase in longevity worldwide requires a greater need for care for older adults, including during the period of hospitalization. In Brazil, there is a growing demand placed on families, who traditionally provide the necessary assistance to older adults unable to carry out daily activities. Hospitalization of older adults constitutes a therapeutic intervention where the family caregiver plays an essential role, contributing to continuity of care and long-term recovery [15].

In this study, there was a predominance of women with an average age of 46.9 years, indicating a higher prevalence of female caregivers. A similar result was found in a study carried out with family caregivers in Latin America [12,13] and the United States [16]. The presence of young adult caregivers poses a challenge, as their functional reserve may be compromised, impacting their ability to perform caregiving tasks effectively. This situation could compromise the quality of care provided to the most dependent older adults [12].

Regarding the relationship with the older adults being cared for, it was evident that the daughters took over the care, confirming that the act of caring was assigned to the woman. Historically, women have taken the task of caregiving as another role within the domestic sphere [12]. Studies indicate that in Canada [17], Europe [18], and the United States [16], family caregivers struggle for social recognition and economic support from the state to fulfill their role; there is a worldwide invisibility of the work of women caregivers, which impoverishes them financially while enriching the capitalist and patriarchal systems.

Regarding marital status, the majority of family caregivers were single, which contrasts with a national study conducted in Brazil that showed a predominance of married caregivers [19]. Single women often play the role of informal caregivers, mainly because they do not have an established family, making them more available to care for their parents. Single women seem to be, in addition to being more available, more pressured by family members to perform this role [20].

In terms of education, the study showed that the majority of caregivers had completed secondary education. Education can influence the understanding of the care to be implemented during the older adults’ hospitalization period as well as the guidelines for preparing for hospital discharge. Therefore, health professionals must be aware of the resources adopted in light of the guidelines given to family caregivers, so that possible mistakes are prevented [19,21].

Regarding the self-reported health condition, the majority of family caregivers reported it as good or neither good nor bad and stated that their health status was not affected by the provision of care. It is possible that the mild degree of dependence of older adults can provide greater peace of mind and less damage to the caregivers’ physical and emotional health [22]. In this case, health professionals must help prevent diseases and promote health for informal caregivers, taking into account their specificities [23].

In the context of providing care, most people regularly care for a person. This may lead to an overload and adversely affect the caregiver’s quality of life, especially when performing the caregiving tasks alone over an extended period of time. The Brazilian Classification of Occupation under code 5162 defines a caregiver as someone who provides care based on the objectives established by specialized institutions or those directly responsible, ensuring well-being, health, nutrition, personal hygiene, education, culture, recreation, and leisure of the assisted person. It is a person, from the family or community, who provides care to other people of any age, who are in need of care due to being bedridden, with physical or mental limitations. An informal/family caregiver is defined as someone who provides care without remuneration, and a formal caregiver is defined as someone who provides care with remuneration and in a professional category. It is worth highlighting the importance of the nurse’s role in establishing a bond with the informal caregiver to carry out health education, a necessary tool in this context [24].

Regarding the profile of hospitalized older adults, the majority were men, a result that corroborates a study carried out with hospitalized Brazilian older adults. The male gender’s tendency to neglect their health can contribute to an increased likelihood of hospital admissions [24].

The average age of people in need of care was 69 years, with a lower degree of dependence in the age group between 60 and 69 years. In another research carried out with hospitalized older adults, it was revealed that older adults’ age is directly linked to the decline in functionality, making them fragile, with a predisposition to falls, presence of dizziness, and some degree of dependence for activities of daily living, directly affecting their autonomy [25].

In this study, we highlighted the association between self-assessment and demographic data (ie, gender, age, and education). Our results align with findings from a previous study on Brazilian family caregivers, which showed that female gender and younger age are associated with greater vitality among family caregivers, a role most often carried out by daughters [10].

Regarding functionality, according to the Barthel Index, there was a predominance of hospitalized older adults with mild dependence, corroborating studies conducted with hospitalized older adults in Brazil [4,25]. Aging is marked by diseases that can have repercussions on functionality, representing a challenge for public health. In this context, health professionals must promote actions that enable the prevention of disabilities, in addition to actions promoting and maintaining the health of older adults, enabling their functional autonomy.

The limitations of the study were the scarcity of research on family caregivers of hospitalized older adults using the Barthel scale, specifically in the Amazon region, which makes comparison with other statistical data difficult. Another limitation was the cross-sectional nature of this study, which means that longitudinal studies may produce different or more precise results, in addition to investigating the impact and influence of the caregiver’s demographic data in relation to the functional capacity of older adults.

The study contributes, therefore, by providing information on the demographic and health profile of family caregivers. These data can help health professionals in planning more individualized assistance, thereby improving quality of life and reducing the impact of the illness. Additionally, the study contributes to actions that promote the functional capacity of older adults, enabling the development of prevention and health promotion strategies. These efforts aim to ensure that the principles of the Unified Health System—equity, integrality, and universality—can be experienced in the act of care.

### Conclusions

The results of this study revealed that family caregivers are mostly women with an average age of 46.9 years, single, and with complete secondary education. The majority self-assessed their health as good, suggesting that the act of caring does not interfere with their ability to provide care, which was normally for their parents with a mild degree of dependence. It is noteworthy that there is an association between gender, age, and education level and the family caregiver’s self-assessment of health.

This study contributes to the filed by addressing the need for research in the hospital context, promoting actions that can implement the provision of care aimed at the autonomy of hospitalized older adults and the development of a hospital assessment to assist family caregivers in their care for the older adults.

## Abbreviations

HUJBB: João de Barros Barreto University Hospital Complex
STROBE: Strengthening the Reporting of Observational Studies in Epidemiology

## Data Availability

The data sets used and analyzed during this study are available upon reasonable request to the corresponding author.
